# Como Saber se uma Mudança é uma melhoria? O (Não Tão) Novo Conhecimento Científico que todo Médico deve Aprender, Dominar e Liderar

**DOI:** 10.36660/abc.20200249

**Published:** 2020-05-12

**Authors:** Alexandre Siciliano Colafranceschi

**Affiliations:** 1 Universidade Federal do Estado do Rio de Janeiro Rio de Janeiro RJ Brasil Universidade Federal do Estado do Rio de Janeiro,Rio de Janeiro, RJ - Brasil; 2 Instituto Nacional de Cardiologia Rio de Janeiro RJ Brasil Instituto Nacional de Cardiologia,Rio de Janeiro, RJ - Brasil; 3 Hospital Pró Cardíaco Rio de Janeiro RJ Brasil Hospital Pró Cardíaco, Rio de Janeiro, RJ – Brasil

**Keywords:** Procedimentos Cirúrgicos Cardiovasculares/tendências, Melhoria da Qualidade, Segurança do Paciente, Mortalidade Hospitalar, Banco de Dados

Os cirurgiões cardiotorácicos têm uma rica história de melhora da qualidade e um forte caráter de transparência e inovação, permitindo a rápida difusão de padrões, técnicas e referências em todo o mundo. A nível nacional, poucas especialidades médicas contribuíram tanto para o desenvolvimento do conhecimento quanto a cirurgia cardíaca brasileira. Desde o trabalho intenso desenvolvido por décadas por cirurgiões pioneiros como Euryclides Zerbini e Adib Jatene até os líderes mais contemporâneos da área, o Instituto do Coração do Hospital das Clínicas da Faculdade de Medicina da Universidade de São Paulo – InCor – está definitivamente no coração desta jornada.^1^ Nesta edição da Arquivos Brasileiros de Cardiologia, o trabalho de Mejia et al,.^2^ tem o mérito de levar em consideração a evolução do número de cirurgias cardiovasculares realizadas no InCor durante um período de 35 anos. O número total é notável: foram analisados mais de 100.000 procedimentos de coração aberto. Afinal, o número médio de procedimentos/ano é 2.964, – ou mais de 11 procedimentos por dia de trabalho. Destaca-se o fato de o número total de procedimentos estar aumentando, principalmente devido ao aumento das cirurgias valvares e à correção das cardiopatias congênitas. Além disso, há uma redução de 7% no volume de cirurgias de revascularização do miocárdio no mais recente período estudado.

Além de descrever o volume de procedimentos cirúrgicos de diferentes doenças cardiovasculares ao longo de cinco períodos diferentes durante os 35 anos de dados, outro objetivo do estudo de Mejia et al.,^2^ foi avaliar o impacto das ações realizadas de um programa de melhoria contínua da qualidade na mortalidade por cirurgia cardiovascular. Não está claro, no entanto, como os períodos foram selecionados para as análises.

A iniciativa de melhora da qualidade no InCor, denominada “Programa de Melhora Contínua da Qualidade” (PMCQ), foi consolidada em 2016 com uma clara missão de diminuir a mortalidade cirúrgica operatória cardiovascular. Ela faz parte da Unidade Cirúrgica de Qualidade e Segurança do Paciente Cirúrgico (UCQSP) como um departamento da Divisão Cirúrgica Cardiovascular do InCor. De acordo com os autores, esta unidade visa apoiar a construção da cultura de segurança, promover a transparência, padronizar o treinamento, melhorar o trabalho das equipes e monitorar o desempenho cirúrgico.^2^

Quando o InCor visa apoiar a construção de uma cultura de segurança, fica claro que eles estão na direção certa. Como afirma Robert Lloyd,^3^ vice-presidente do *Institute for Healthcare Improvement* , “Qualidade” não é um departamento. Uma organização só fará melhoras significativas e sustentáveis quando as pessoas em todos os níveis sentirem um desejo e uma responsabilidade compartilhados para melhorar processos e resultados todos os dias.

Após a análise dos dados, os autores concluíram que houve uma diminuição significativa da mortalidade operatória (mais próxima dos padrões internacionais) nos grupos estudados após a implementação do programa de melhora da qualidade no InCor. A questão que permanece é, como sabemos que as alterações feitas após a consolidação do PMCQ resultaram em uma melhoria na mortalidade cirúrgica?

Dirigir esforços na coleta, análise e aplicação de dados dos resultados cirúrgicos a fim de melhorar a qualidade e reavaliar condutas e procedimentos é fundamental para as iniciativas de melhora da qualidade. Entretanto, misturar medidas de prestação de contas ou pesquisa com medidas de melhora é contraproducente.^4^

Os conceitos modernos de Melhora de Qualidade (QI, *Quality Improvement* ) tiveram sua origem nas medidas do Controle Estatístico de Processo (CEP), desenvolvidas por Walter Shewart na década de 1920. O casamento dessas técnicas com uma filosofia de gerenciamento geral de Edwards Deming, Joseph Juran e outros resultou no movimento da qualidade, conhecido por vários termos e acrônimos (TQM - Total Quality Management, CQI - *Continuous Quality Improvement* , etc.). Embora tenham chegado mais tarde à área de cuidados médicos do que em outros campos, os conceitos de QI proliferaram rapidamente aqui através dos esforços de Berwick e outros.^5^

A melhora da qualidade requer o uso de dados para aprender e prever o desempenho futuro (em oposição ao que aconteceu no passado, conforme revelado pelos dados de prestação de contas e pesquisa). Em relação à melhora, é fundamental entender que todo processo tem uma variação inerente que se deseja entender. A compreensão dos termos processo e variação, além de desenvolver o pensamento do processo, é fundamental para entender como melhorar alguma coisa.

O cuidado cirúrgico cardiotorácico contemporâneo é um processo complexo, envolvendo técnicas e equipamentos sofisticados, profissionais de saúde com níveis variados de capacidade e pacientes de alto risco. Os cirurgiões trabalham em ambientes críticos de segurança, onde a complexidade do cuidado e os fatores de risco dos pacientes aumentam exponencialmente o potencial para danos significativos. O sistema projetado de cuidado a pacientes cirúrgicos fornece resultados que variam ao longo do tempo, independentemente de serem bem-sucedidos ou não. Como seres humanos e sistemas mal projetados são vulneráveis a erros, uma avaliação crítica de nossos sistemas de cuidado é essencial para que a melhora continue.^6^

A variação em uma medida de qualidade pode resultar de *causas comuns* –causas esperadas que são inerentes ao sistema. Também pode ser derivada de *causas especiais* – causas não naturais que não fazem parte do sistema, mas surgem devido a circunstâncias específicas.

**Figura 1:**
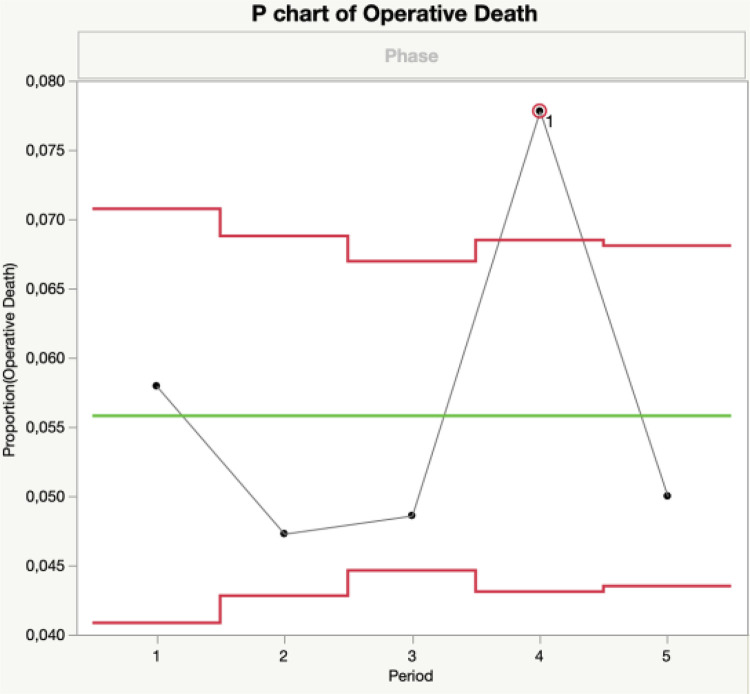
Gráfico P de morte operatória. Dados obtidos das tabelas 1 e 2 do manuscrito original. A linha média está em verde. Os limites de controle superior e inferior estão em vermelho. Os pontos representam o índice de óbito operatório para o período especificado. Os pontos são conectados em uma linha preta que mostra variação. A morte operatória no período 4 está além do limite superior de controle (como marcado em um círculo vermelho), sugerindo uma causa especial no processo de cuidado nesse período de coleta de dados.

Existem muitas maneiras de apresentar e analisar dados. Para os esforços de melhoria, um *gráfico de controle* ( Figura1 ) ajuda a distinguir entre causas comuns e especiais de variação. Ele inclui um limite de controle superior e um de controle inferior marcado acima e abaixo da linha média. Variações dentro desses limites são esperadas e atribuídas a causas comuns; a variação além desses limites sugere causas especiais.^7^

Em um sistema estável, apenas causas comuns afetam os resultados. A variação é previsível dentro dos limites estatisticamente estabelecidos. Em contraste, em um sistema instável, os resultados são afetados por causas comuns e causas especiais. Nesse caso, a variação é imprevisível. Se o processo é estável e a variação é previsível, é possível prever o resultado futuro para o sistema que está sendo observado em tempo real, o que o torna adequado para os esforços de melhoria. Os *gráficos de controle* também podem ser utilizados para identificar sinais precoces de sucesso em um projeto de melhoria e monitorar um processo para garantir que ele esteja mantendo os ganhos advindos de um esforço de melhora da qualidade. Como um *gráfico de execução* , ajuda a determinar se as alterações feitas estão levando a melhoras. O ponto aqui é que os esforços de melhoria só podem ser feitos em sistemas estáveis.^7^

Os dados extraídos do manuscrito original em suas tabelas 1 e 2 (volume total de procedimentos e índice total de óbitos operatórios ao longo de diferentes períodos de tempo) foram utilizados para criar um gráfico de controle, como na Figura 1 . As técnicas de controle estatístico de processo (CEP) oferecem um papel eficaz no monitoramento do desempenho hospitalar, como taxa de mortalidade.^6^ De acordo com essa análise, o sistema sendo utilizado para esforços de melhoria no trabalho de Mejia et al.,^2^ é um sistema instável e o resultado (morte operatória) é afetado por causas comuns e especiais. Como a variação é imprevisível em um sistema instável, as alterações do PMCQ no InCor não podem ser atribuídas à melhora na mortalidade operatória total do período 4 para o período 5. De fato, utilizando a metodologia CEP, não há diferença na mortalidade operatória entre os períodos 1, 2,3 e 5. A mortalidade operatória no InCor tem variado próximo aos padrões internacionais desde que começaram a coletar esses dados. Uma causa especial no período 4 aumentou a mortalidade operatória além do limite superior de controle, o que a tornou estatisticamente diferente do período 5, quando métodos estatísticos de pesquisa foram utilizados para analisar um esforço de melhoria.

As organizações de saúde utilizam os dados para entender seu desempenho – embora nem sempre o façam com eficácia.^4^ É importante observar que a equipe de melhoria da qualidade vizualiza e busca utilizar dados referentes à variação nos processos de assistência à saúde de maneira diferente daquela dos pesquisadores de serviços de saúde. Onde a melhoria da qualidade prática e em tempo real é a meta, a própria variação precisa ser examinada em tempo real para responder às perguntas: 1- Estamos melhorando? E 2- Onde podemos melhorar?^4^ Assim, os dados de desempenho “ *just-in-time* ” são essenciais para o uso efetivo dos dados de variação, e o foco está na criação de processos estáveis e no aprendizado de variações por causas especiais. Por outro lado, os pesquisadores de serviços de saúde levantam a questão: A causa B? (se as outras coisas forem iguais), muitas vezes considerando a visão de longo prazo para examinar dados de vários anos e buscando eliminar variações de causas especiais e testar a significância.^8^ Essas diferentes perspectivas podem levar administradores e pesquisadores da saúde a olharem para os mesmos resultados e chegarem a conclusões muito diferentes sobre sua significância e as ações que devem ser tomadas em resposta.^4^

Aprender rapidamente com os erros faz parte das teorias da melhoria e, embora não tenha sido comprovada a melhora na mortalidade operatória atribuída às ações tomadas após a consolidação do PMCQ, o esforço contínuo de melhoria da qualidade no InCor está longe de ser malsucedido. A iniciativa do PMCQ no InCor deve ser seguida por outros. O InCor não apenas foi pioneiro e dominou a academia de cirurgia cardíaca no país. Sua liderança no campo continua a mudar nossas próprias perspectivas sobre o que significa ser um cirurgião cardíaco contemporâneo dentro de um sistema. O InCor está nos ajudando a refletir sobre a visão tradicional de que os resultados dos pacientes estão relacionados apenas à habilidade técnica do cirurgião para uma estrutura em evolução e mais ampla, na qual os resultados da assistência à saúde são afetados por uma infinidade de fatores em processos e ambiente altamente integrados e complexos. Como médicos (e cirurgiões) estão envolvidos em quase todos os processos importantes de assistência à saúde, é um desperdício tentar melhorar os processos de assistência à saúde sem eles.^5^ Ainda é necessário que o cirurgião aprenda, domine e lidere a incorporação de novas tecnologias e as habilidades técnicas para cuidar dos pacientes. Contemporaneamente, entretanto, isso não é suficiente para melhorar os resultados. Chegou a hora dos cirurgiões cardiotorácicos (e de todos os médicos) refletirem sobre seus próprios propósitos pessoais de serem profissionais de saúde e aprenderem, dominarem e liderarem o (não tão) novo conhecimento científico para melhoria dos processos assistenciais dos pacientes.
