# Supplementary material for: Como Saber se uma Mudança é uma melhoria? O (Não Tão) Novo Conhecimento Científico que todo Médico deve Aprender, Dominar e Liderar
Source: Arq Bras Cardiol. 2020 May 12;114(4):613–5. [Article in Portuguese] doi: 10.36660/abc.20200249 (PMC9744339; doi:10.36660/abc.20200249)
Supplement: Translation

# Translation: How Do We Know a Change is an Improvement? The (Not So) New Scientific Knowledge Every Physician Should Learn, Master and Lead

**Authors:** Alexandre Siciliano Colafranceschi

**Affiliations:** 1 Brazil Universidade Federal do Estado do Rio de Janeiro, Rio de Janeiro, RJ - Brazil; 2 Brazil Instituto Nacional de Cardiologia, Rio de Janeiro, RJ - Brazil; 3 Brazil Hospital Pró Cardíaco, Rio de Janeiro, RJ – Brazil

**Keywords:** Cardiovascular Surgical Procedures/trends, Quality Improvement, Patient Safety, Hospital Mortality, Database

Cardiothoracic surgeons have a rich history of quality improvement and a strong ethos of transparency and innovation allowing for the rapid diffusion of standards, techniques, and benchmarks worldwide. Nationally, few medical specialties have contributed as much to the development of knowledge as the Brazilian cardiac surgery. From the hard work developed during decades by pioneering surgeons such as Euryclides Zerbini and Adib Jatene to the most contemporaneous leaders in the field, the *Instituto do Coração do Hospital das Clínicas da Faculdade de Medicina da Universidade de São Paulo* – InCor – is definitely at the heart of this journey.^1^ In this issue of *Arquivos Brasileiros de Cardiologia* the work by Mejia et al.^2^ has the merit of taking into account the evolution of the number of cardiovascular surgeries performed at InCor during a 35-year period. The total number is a remarkable one: over 100,000 open heart procedures were analyzed. After all, the mean number of procedures/year is 2,964, - or more than 11 procedures per workday. It is noteworthy the fact that the total number of procedures has been increasing, especially due to an increase in valvular operations and the correction of congenital cardiopathies. Also, of note, there is a 7% decrease in coronary artery bypass graft surgery volume in the most recent period studied.

Besides describing the volume of disease-specific open-heart surgical procedures throughout five different periods of time over the 35 years of data, another objective of the study by Mejia et al.^2^ was to evaluate the impact of the actions taken from a continuous quality improvement program on mortality from cardiovascular surgery. It is not clear, however, how the periods of time were selected for the analyses.

The quality improvement initiative at InCor, called *Programa de Melhoria Contínua da Qualidade (PMCQ),* was consolidated in 2016 with a clear mission to decrease the cardiovascular surgical operative mortality. It is hosted at the *Unidade Cirúrgica de Qualidade e Segurança do Paciente Cirúrgico (UCQSP)* as a department of the Cardiovascular Surgical Division at InCor. According to the authors, this unit aims to support the construction of the safety culture, promote transparency, standardize training, improve the work of the teams and monitor the surgical performance.^2^

When InCor aims to support the construction of a safety culture, it is clear that they head toward the right direction. As stated by Robert Lloyd,^3^ Vice President at the Institute for Healthcare Improvement, “Quality” is not a department. An organization will only make meaningful and sustainable improvements when people at every level feel a shared desire and responsibility for making processes and outcomes better every day.

After analyzing the data, the authors concluded that there was a significant decrease in operative mortality (closer to international standards) in the studied groups after the implementation of the quality improvement program at InCor. The question that remains is how do we know that the changes made after the PMCQ consolidation resulted in an improvement in surgical mortality?

Directing efforts in collecting, analyzing and applying data of the surgical results in order to improve quality and reassess conducts and procedures is critical to quality improvement initiatives. Mixing accountability or research measures with those for improvement, however, is counterproductive.^4^

Modern Quality Improvement (QI) concepts had their origins in the Statistical Process Control (SPC) measurements developed by Walter Shewart in the 1920s. The marriage of those techniques with an overall management philosophy by Edwards Deming, Joseph Juran, and others has resulted in the quality movement as it is known through various terms and acronyms (TQM – Total Quality Management, CQI – Continuous Quality Improvement, and so on). Although arriving later in health care than in other fields, QI concepts have rapidly proliferated here through the efforts of Berwick and others.^5^

Quality improvement requires using data to learn and to predict future performance (as opposed to what happened in the past, as stated by accountability and research data). Regarding improvement, it is critical to understand that every process has an inherent variation that one wants to understand. Understanding the terms process and variation, besides developing process thinking, are fundamental to an understanding of how to improve anything.

Contemporary cardiothoracic surgical care is a complex process, involving sophisticated techniques and equipment, health care professionals with varying levels of skills, and high-risk patients. Surgeons work in a safety-critical environments where the complexity of care and the patients’ risk factors exponentially increase the potential for significant harm. The designed system of caring for surgical patients deliver outcomes that vary throughout time, irrespective of being successful or not. Because humans and poorly designed systems are vulnerable to error, a critical assessment of our systems of care is essential for improvement to continue.^6^

Variation in a quality measure may result from *common causes* — expected causes that are inherent to the system. It may also derive from *special causes* — unnatural causes that are not part of the system but arise due to specific circumstances.

There are many ways to present and analyze data. For improvement efforts, a *control chart* ( Figure 1 ) helps distinguish between special and common causes of variation. It includes an upper control limit and a lower control limit marked above and below the average line. Variation within these limits is expected and attributed to common causes; variation beyond these limits suggests special causes.^7^

**Figure 1:** P chart of operative death. Data obtained from tables 1 and 2 of the original manuscript. Average line is in green. Upper and lower control limits are in red. Dots represents the operative death index for the specified period of time. Dots are connected in a black line that shows variation. Operative death in period 4 is beyond the upper control limit (as marked in a red circle), suggesting a special cause in the process of caring within this period of data collection.

In a stable system, only common causes affect the outcomes. Variation is predictable within statistically established limits. By contrast, in an unstable system, outcomes are affected by both common causes and special causes. In this case, variation is unpredictable. If the process is stable and variation is predictable, one can foresee the future outcome for the system being observed in real-time, which makes it suitable for improvement efforts. *Control charts* can also be used to identify early signs of success in an improvement project and to monitor a process to ensure it is holding the gains from a quality improvement effort. Like a *run chart* it helps determine whether the changes made are leading to improvement. The point here is that improvement efforts can only be made in stable systems.^7^

Data driven from the original manuscript in its tables 1 and 2 (total volume of procedures and total operative death index throughout different periods of time) were used to build a control chart as in Figure 1 . Statistical process control (SPC) techniques have played an efficacious role in monitoring hospital performance, such as mortality rate.^6^ According to this analysis, the system being used for improvement efforts in the work by Mejia et al.^2^ is an unstable system and the outcome (operative death) is affected by both common and special causes. Since variation is unpredictable in an unstable system, the changes from PMCQ at InCor cannot be attributed to the improvement in total operative death from period 4 to period 5. In fact, using SPC methodology, there is no difference in operative mortality between periods 1,2,3 and 5. The operative mortality at InCor has been varying close to international standards since they started collecting these data. A special cause in period 4 increased the operative mortality beyond the upper control limit, which made it statistically different from period 5, when research statistical methods were used to analyze an improvement effort.

Health care organizations use data to understand their performance — although they do not always do so effectively.^4^ It is important to note that the quality improvement staff view and seek to use data regarding variation in healthcare processes differently from that of health services researchers. Where practical, real-time quality improvement is the goal, variation itself needs to be examined in real time to answer the questions: 1- Are we getting better? And 2- Where can we improve?^4^ Thus, “just-in-time” performance data are essential to the effective use of variation data, and the focus is on creating stable processes and learning from special-cause variation *.* In contrast, health services researchers pose the question, does A cause B (other things being equal?), often taking the long view to examine several years` worth of data and seeking to eliminate special-cause variation and test for significance_._^8^These different perspectives can lead healthcare managers and researchers to look at the same results and reach very different conclusions about their significance and the actions that should be taken in response.^4^

Learning fast from mistakes is part of the improvement theories and although there was no proven improvement in operative mortality attributed to actions taken after PMCQ consolidation, the continuous quality improvement effort at InCor is far from being unsuccessful. The PMCQ initiative at InCor should be followed by others. InCor not only pioneered and mastered the open-heart surgical academy in the country. Its leadership in the field continues to change our own perspectives regarding what means to be a contemporary heart surgeon within a system. InCor is helping us reflect on the traditional view that patient outcomes are related only to the surgeon’s technical skill to an evolving and broader framework, wherein health care outcomes are affected by a multitude of factors in highly integrated and complex processes and environment. Since physicians (and surgeons) are involved in almost all-important health care processes, it is wasteful to try to improve health care processes without them.^5^ It is still required for a surgeon to learn, master and lead the current and new technology and technical skills to care for patients. Contemporarily, however, this is not sufficient to improve outcomes. It is time for cardiothoracic surgeons (and every physician) to reflect on their own personal purposes of being a healthcare professional and learn, master and lead the (not so) new scientific knowledge to improve patient outcomes.
